# Ceramide in Cystic Fibrosis: A Potential New Target for Therapeutic Intervention

**DOI:** 10.1155/2011/674968

**Published:** 2010-12-28

**Authors:** Gabriella Wojewodka, Juan B. De Sanctis, Danuta Radzioch

**Affiliations:** ^1^Human Genetics, McGill University Health Center Research Institute, 1650 Cedar Avenue L11-218, Montreal, QC, Canada H3G 1A4; ^2^Institute of Immunology, Central University of Venezuela, Apartado Postale 50109, Caracas 1050A, Venezuela; ^3^Departments of Medicine and Human Genetics, McGill University Health Center Research Institute, 1650 Cedar Avenue L11-218, Montreal, QC, Canada H3G 1A4

## Abstract

Patients with cystic fibrosis (CF) are afflicted with many symptoms but the greatest challenge is the fight against chronic bacterial infections, leading to decreased lung function and ultimately death. Our group has recently found reduced levels of ceramides in CF patients and mice. Ceramides are sphingolipids involved in the structure of cell membranes but also participate in the inflammatory response, in cell signalling through membrane microdomains (lipid rafts), and in apoptosis. These characteristics of ceramides make them strong candidates for therapeutic intervention in CF. As more studies have come to evaluate the role of ceramide in CF, conflicting results have been described. This paper discusses various views regarding the potential role of ceramide in CF, summarizes methods of ceramide detection and their role in the regulation of cellular and molecular processes.

## 1. Introduction

Cystic fibrosis (CF) is the most common autosomal recessive disorder found in populations of European descent. It is estimated that 70,000 individuals live with the disease around the world (see http://www.cff.org/AboutCF/) with 70% of patients having the ΔF508 mutation. The disease is caused by a mutation in the Cystic Fibrosis Transmembrane Conductance Regulator (CFTR) gene which encodes for a chloride channel. Mutations in the gene cause decreased chloride transport out of the cell; consequently, there is an increased absorption of sodium and reduction in the airway surface liquid. Mucociliary clearance is thus impaired creating a perfect environment for bacterial colonization. Phenotypes of CF are recurrent and chronic pulmonary infections, decreased lung function, pancreatic insufficiency, diabetes, meconium ileus, infertility in males, osteoporosis, and fatty acid defects [1–4]. 

Current therapies for CF include aerosolized antibiotics such as tobramycin, recombinant human DNase such as dornase alfa for improved mucus clearance, hypertonic saline for increases in airway surface liquid, and nonsteroidal antiinflammatory drugs (NSAIDs) like Ibuprofen to decrease the inflammatory response. Other antibiotics such as azithromycin are commonly used to fight *P. aeruginosa* infections [5]. Bronchodilators are also commonly prescribed to improve breathing [5]. CF patients take a combination of drugs to improve or maintain lung function not to mention vitamins and pancreatic enzymes and, at times, insulin for pancreatic insufficiency [6] and diabetes [7]. Despite prophylactic treatments, CF patients still suffer from chronic infections and exacerbations which have psychological impacts and ultimately impact the progression of the disease. 

Recently, we have described a new characteristic observed in CF patients and mice. Concentrations of ceramide were found to be reduced in plasma of CF patients and mice as well as CF-related organs in mice: lungs, pancreas, and ileum [8]. Subsequent studies have investigated the importance of ceramide in the context of CF indicating that it might be a potential target for new therapies. However, contrary to our data, certain groups have reported elevated levels of ceramide in CF tissues from patients and mice. These contradicting results have shed light on the potential role of ceramide in CF; however, they also bring attention to the issues surrounding the different methods of ceramide analysis. This paper will examine why ceramide should be considered a key player in the symptoms of CF disease. Importance will be placed on the methodologies used to analyze ceramide and also the various models used to study ceramide in CF. 

## 2. Ceramides

### 2.1. Roles, Synthesis, and Structure

Ceramides are molecules from the sphingolipid family. They are known to be involved in membrane rigidity and permeability, apoptosis and can act as second messengers signalling to Cathepsin D, PKC, PP2A, and c-Raf [9]. In the cell membrane, ceramides are components of microdomains (lipid rafts) which are important in transmembrane signalling [10]. 

**Figure 1:**
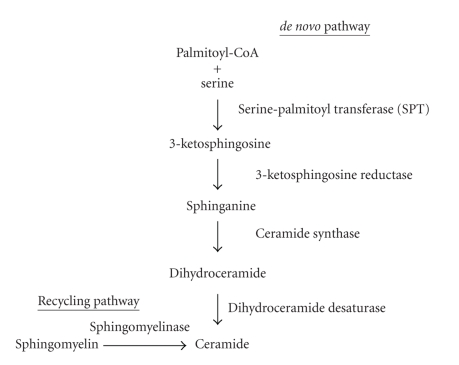
Synthesis pathway of ceramide. There exist two pathways for ceramide synthesis: *de novo* pathway and recycling pathway. The *de novo* pathway occurs in the endoplasmic reticulum while the recycling pathway occurs in vesicular and cell membranes.

There are two pathways for ceramide synthesis (Figure 1). The *de novo* pathway occurs in the endoplasmic reticulum and begins with the conversion of serine and palmitoyl-CoA to sphinganine by serine palmitoyl transferase. Ceramide synthases then transform sphinganine into dihydroceramide which is converted to ceramide by dihydroceramide desaturase. Alternatively, ceramide can be synthesized through a recycling pathway with the conversion of sphingomyelin to ceramide by sphingomyelinase (SMase) [9]. There are several types of SMase categorized based on pH optima and localization in the cell: acidic (aSMase), neutral (nSMase), and alkaline (Alk-SMase) [11]. Generally a pH of 4.5–5.0 is optimal for aSMase and it is localized mainly in lysosomal compartments in the cells as well as in membrane microdomains (discussed later) in the cell membrane [12]. Neutral SMases have a pH optima of 7.4 [12] and can be located in the plasma membrane [13]. Alk-SMase is mainly found to be expressed in the intestines [14]. Considering that the deficiency in ceramides in CF can be corrected by fenretinide and the induction of dihydroceramide, the precursor to ceramide, can be detected [8], these findings suggest that the *de novo* pathway involving the endoplasmic reticulum is targeted by this drug in CF as it was described before in the context of cancer. 

Ceramides are defined by the presence of a sphingoid base in their backbone. A sphingoid base is the name given to the carbon chain containing 2 to 3 OH groups and often a double bond at carbon 4 present in sphingolipids [9]. All ceramides have a sphingoid backbone of 18 carbons; however, they can differ in the number of carbons in the fatty acid moiety. The sphingoid backbone contains a double bond in ceramides which when removed forms dihydroceramide (DHC) often indistinguishable from ceramide when using methods based on the analysis of total ceramide levels [15]. Ceramides can also have other molecular modifications such as the addition of glucose to obtain glucosylceramide and galactose to have galactosylceramide [9]. These molecules are also metabolically active and while not specifically analyzed in the studies that will be later described later on, they remain important members of the sphingolipid family.

### 2.2. Detection of Ceramides

There are several issues to consider when developing an appropriate methodology for the analysis of ceramides. Phospholipids constitute approximately 30% of lipids in cells [16]. These molecules are similar in structure thus the analysis requires high level of specificity. Ceramides alone represent only 1% of lipids present in the cell [15]. Therefore, a sensitive and highly specific technique must be utilized for an accurate analysis. 

#### 2.2.1. Diacylglycerol Kinase Assay

One of the most common biochemical assays for ceramide detection involves the use of diacylglycerol (DAG) kinase which can convert ceramide to ceramide-1-phosphate. The assay quantifies levels of ceramides by incubating extracted lipids with [P^32^] ATP and DAG kinase and assessing the levels of [P^32^] ceramide-1-phosphate. There are certain caveats with this method [17], and there has been some question regarding the validity of the results generated with this assay [18, 19]. The source and amounts of the enzyme and ATP can alter the results obtained. Additionally, an internal standard should ideally be used to normalize the level of enzyme activity [15]. In summary, proper care must be taken into consideration to produce valid results [20], and ideally data should be verified using a more sensitive technique.

#### 2.2.2. Immunodetection

There exist two main antibodies which have been employed in ceramide detection. A monoclonal antibody clone 15B4 (Sigma-Aldrich, Oakville, Ontario, Canada) was employed by our group using lipids purified by thin layered chromatography (TLC) in an ELISA assay [8] and by others in immunohistochemistry [21]. A polyclonal antibody, IgM-enriched mouse anticeramide antiserum (Glycobiotech, Kükels Germany, formerly identified as MAS 0010) and more recently a monoclonal anticeramide antibody (S58-9, same molecule as MAS 0010 sold without the addition of mouse serum, Glycobiotech), has been used by some CF investigators [21, 22]. 

Both antibodies were evaluated and compared in a study by Cowart and colleagues [23]. The study tested the affinity of the antibodies to specific species of ceramide using lipid overlay assays and it was found that each antibody recognizes different species and both recognize dihydroceramide. The monoclonal antibody (15B4) had stronger affinities to C14 and C24 ceramide than the polyclonal. It also could recognize C16 albeit giving a weaker signal than the polyclonal antibody. It also recognized phosphatidylcholine. The polyclonal antibody had a strong affinity to C14 and C16 ceramides but had low detection of C10 ceramide. No other cross-reactivity was observed in this study using lipid overlay assays [23]. The Cowart study observed that variable results can be obtained when using different techniques to evaluate antibody sensitivity. Depending on the epitope exposure, the antibodies may also cross-react with other species of sphingolipids. For instance, the authors specify that they did not compare the antibodies in the context of ELISAs and suggest that the recognition of ceramide species may differ using this method [23]. Another study was conducted to specifically characterize the polyclonal antibody. The results of dot blot assays showed that the antibody was specific to long-chain ceramides found in the skin (ceramide-2 [N-acylsphingosine], ceramide-3 [N-acylphytosphingosine], ceramide-5 [N-(alpha-hydroxy)acylsphingosine] [24] ), C14 and C16 ceramides, and C16-dihydroceramide [25]. Cholesterol could also be detected with the antibody when present in high concentrations in thin-layered chromatography assays. Dot blot assays were used to characterize the monoclonal antibody and it was found to detect weakly C16-dihydroceramide and strong sphingomyelin [25]. Affinities to ceramides besides ceramide-1 ([N-(omega-acyloxy) acylsphingosine] [24]), ceramide-2, ceramide-3 and ceramide-5, C14, C16, and C16-dihydroceramide were not assessed with the monoclonal antibody [25]. 

The studies using antibodies in ceramide quantification have used them in different ways. Our group has used the monoclonal antibody in ELISAs only after the ceramide fraction was isolated from lipids using thin layered chromatography [8]. Other studies have used these antibodies for immunohistochemistry detecting the amount of staining for a designated area with computer software [21, 22]. 

In sum, data obtained using antibodies must be critically evaluated and should be verified with the methods able to assess the complete spectrum of detectable ceramide species and not only selected species of ceramides. 

#### 2.2.3. Mass Spectrometry

With the development and refinement of separation techniques such as liquid and gas chromatography (LC and GC) and mass spectrometry (MS), an accurate analysis of ceramides is now possible. Using an appropriate ionization technique and lipid extraction methods, it is feasible to determine not only the concentrations of ceramides in a sample, but also to quantify the amounts of the numerous ceramide species during each analysis [16, 26–28]. High pressure LC (HPLC) is often used as a primary separation of lipids extracted from a given sample. Lipid species of similar molecular structure will elute off the column at similar times. Upon entry into the mass spectrometer, the electrospray ionization (ESI) method is used to ionize molecules. It is a soft ionization technique which is not likely to cause damage to the lipid molecules and is currently the favoured method of lipid ionization [29]. Finally, to distinguish between the various ceramide species, the molecules undergo two MS analyses (tandem MS or MS/MS) where molecules are selected based on their mass to charge ratio or *m/z*. There are a few techniques used to determine amounts of ceramide at this step. 

The product ion analysis method involves the detection of molecules of a specific *m/z *in the first MS step (occurring in the first quadrupole or Q1). The molecules selected will undergo fragmentation by gas collision in Q2. Finally in the second MS step, the fragments generated will be analyzed in Q3 which will scan different *m/z *settings resulting in a spectrum of fragments issuing from the precursor ions selected for in Q1 [28]. 

The precursor ion analysis is the opposite of product ion scan. The Q1 is set to scan a range of *m/z*, 500 to 670 *m/z,* to select for all ceramide species [27]. These molecules are fragmented in Q2 and specific fragments are detected in the second round of MS in Q3. Ceramide molecules will be fragmented into two distinct parts: the sphingoid backbone and the carbon chain of varying lengths [27]. In Q3, the detector is set at 264 *m/z* and will only detect the sphingoid backbone from the fragments generated in Q2. To analyse sphingoid backbones of dihydroceramides, the detector must be set at 266 *m/z* [28]. 

Multiple reaction monitoring is an alternative to both methods. Both Q1 and Q3 are set to detect specific *m/z*. Only molecules which pass the specific settings will be detected in the end. However, the analysis is not limited to only one pair of precursor and product ions. The detection can be repeated in a cycle which allows for more sensitive and rapid quantifications [28]. 

There are a few issues to consider when using the MS/MS technique. The most important step is the purification of lipids from the biological samples. Current methods use the procedure established by Folch et al. [30] and modified later by Bligh and Dyer [31] which use a 2 : 1 chloroform/methanol solution for extraction. Appropriate internal standards should be used which will elute from the LC columns and become ionized in a similar way as the ceramides molecules. These standards should be ceramides that do not occur naturally in biological samples such as C12 [28] or a cocktail of standards (Avanti Polar Lipids, Alabaster, AL, USA). To accurately quantify the amounts of different ceramide species, standard curves must be generated and to be precise, one standard curve per analyte should be generated [32]. The concentration of lipids in the sample prior to analysis is also important to prevent lipid aggregation which can occur when lipid concentrations are high. The concentration of lipids is suggested to be below 100 pmol of total lipids/*μ*L [32].

#### 2.2.4. Sphingolipidomics

The previous techniques in MS/MS are incredibly accurate; however, they can only detect known molecules. More recently, researchers have increased their interest in assessing the full spectrum of lipids in a sample. To this end, sphingolipidomics utilizes a shotgun approach. Lipids are extracted to which is added an internal standard for each lipid class. The lipid extraction from samples is further refined so that specific lipid classes can be isolated even before separation methods come into play. The acidic and basic properties of lipids are used to ionize to either positive or negative ions. This step called intrasource separation replaces the separation using LC in the previous MS/MS methods. Before quantitation can occur, a fragment ion unique to a group of lipids of interest must be found to be able to set the detector, for example, 264 *m/z* for ceramides. Finally, the lipids of a specific group can be quantified by comparing to the internal standard for that group [32]. 

There are a few issues that have arisen with this method, mainly that quantification of lipids in low concentrations can be compromised if signals overlap with those from more abundant lipids. There is a lack of internal standards at the moment for certain classes of lipids, which will limit the power of lipidomics for those molecules [32]. Recently, a survey of the lipidome from yeast using shotgun mass spectrometry has revealed that about 95% of the lipidome was covered using this approach [33].

## 3. Ceramide and Bacterial Infections

CF patients are plagued with chronic pulmonary bacterial infections which are the leading cause of mortality in CF. The majority of infections are caused by *Pseudomonas aeruginosa* although *Staphylococcus aureus*, *Haemophilus influenzae*, *and Burkholderia cepacia* are also common pathogens isolated from lungs of patients [34]. There are many factors in CF lungs that facilitate the chronic presence of infections such as increased inflammatory responses, thick layers of mucus, abnormal cell signalling, and defective cell death. There are many instances where ceramide could potentially play a role in CF. 

### 3.1. Ceramide and the Inflammatory Response

CF patients are plagued with high levels of cytokines. In animal models, these levels are triggered despite the absence of infection [35]. It is however unclear whether the abnormal expression of inflammatory genes precedes infection in patients. A few studies have described higher levels of inflammation in infants with CF [36, 37] and one study has described high levels of cytokines in foetuses with diagnosed CF [38]. Hallmarks of increased inflammation in CF are mainly higher levels of IL-1*β* [39], IL-6 [40], IL-8 [41], TNF-*α*, while the antiinflammatory IL-10 was found to be reduced in CF [39]. The transcription factor NF-*κ*B responsible for the transcription of cytokines has increased levels of phosphorylation in CF [42]. CFTR was found to be a negative regulator of NF-*κ*B phosphorylation which would explain higher activation of the transcription factor when CFTR is nonfunctional [43]. 

Ceramides were shown to be generated under certain stimuli such as UV, heat, cytokines, oxidative stress, and LPS and in turn ceramide can regulate the expression of cytokines. A study by Chiba and colleagues revealed that ceramides can have different effects on cytokine expression depending on the cell type. The pretreatment of 10 *μ*g/ml of C8 ceramide reduced the expression of IL-5, IL-10, and IL-13 in mast cells in response to LPS stimulation while TNF*α* and IL-6 levels were unaffected. C8 ceramide did however inhibit TNF*α* and IL-6 in macrophages but did not alter IL-10 levels. In both cell types, the addition of C8 ceramide inhibited the transcription of cytokines in response to LPS; however, the inhibited cytokines were different per cell type [44]. Results comparing cytokine expression between aSMase deficient mice and normal controls showed a 10- to 15-fold increase in serum TNF*α* following LPS administration in aSMase-KO mice. In peritoneal macrophages isolated from aSMase deficient mice, the supplementation with 30 and 60 *μ*M of C2 and C16 ceramide was able to normalize TNF*α* expression following stimulation with LPS [45]. 

IL-8 expression is mainly induced by NF-*κ*B which in turn is activated by TNF*α*, among other mediators, and also involves the activation of the MAPK pathway. Ceramides were shown to activate protein phosphatase 2A (PP2A) [46], a regulator of both NF-*κ*B and MAPK pathways. Using human alveolar epithelial cells, Cornell and colleagues have demonstrated an increase in IL-8 levels when PP2A is inhibited. The authors found that PP2A inhibition increases the signalling through the MAPK pathway by prolonging the activation of JNK, p38, and ERK and enhances the stability of IL-8 mRNA. Similarly, inhibition of ceramide synthesis led to a reduction in PP2A activation and consequently an increase of IL-8 levels. The authors concluded that ceramides are needed to activate PP2A in order to control and, consequently, down regulate expression of IL-8 [47]. 

A recent study demonstrated that activation of p38 MAPK in lungs of CF patients is greater compared to non-CF controls. Following exposure to *P. aeruginosa*, cells expressing the ΔF508 mutation experienced a much greater increase in IL-6 compared to cells expressing normal CFTR due to the overactivation of p38 MAPK [40]. Additionally, decreases in ceramide were found to induce activation of p38 MAPK which resulted in increased IL-6 production. Treatment of alveolar epithelial cells with C6 ceramide prevented the increase in IL-6 [48]. 

 The results of these studies demonstrate the importance of ceramide in regulating excessive inflammatory responses and that a reduction in ceramide levels can result in an uncontrolled production of cytokines which is resolved by supplementation of ceramide. Additionally, the role of the recycling pathway in ceramide generation following LPS stimulation seems to be a key component to the response to infections. 

Azithromycin, a macrolide currently used to treat CF patients, has been shown to improve clinical symptoms of CF such as lung function, improvement in bacterial clearance and attenuation of inflammation. While the mechanism of this drug is still under investigation, it was found recently that it induces expression of certain inflammatory genes and those involved in the lipid pathways. In particular, certain genes involved in the cholesterol pathway were induced. An older study demonstrated that total cell phospholipid content was increased following azythromycin treatment of primary fibroblasts [49] suggesting that manipulation of phospholipids may benefit the outcome of infections in CF.

### 3.2. Ceramide and Signalling Response to *P. aeruginosa*

Signalling following infection occurs mainly through Toll-like receptors (TLR) found in the plasma membrane. TLR-4 is a receptor for lipopolysaccharide (LPS) found on the surface of gram negative bacteria. TLR-4 expression was found to be reduced in CF patients; however when the CFTR defect was corrected in epithelial cells, higher levels of TLR-4 were detected on the cell surface [50]. TLRs are located at the cell surface in lipid rich clusters termed microdomains. Membrane microdomains, also known as lipid rafts, are lipid aggregates in plasma membranes comprised of cholesterol, sphingolipids, and phospholipids. They are involved in signal transduction by forming platforms for receptors activated by their ligands. Microdomains spatially organize proteins and protect them from enzymes which could prevent downstream signalling (e.g., phosphatases) [10]. Kowalski and Pier described the presence of CFTR in membrane microdomains. They postulated that under normal situations, CFTR is a receptor for LPS and induces the internalisation of *P. aeruginosa*, the induction of the inflammatory response, apoptosis and, during infection with *P. aeruginosa,* the presence of CFTR increases in microdomains. The ΔF508 mutation in CFTR prevents the localisation of CFTR into the microdomains and bacterial infection is not cleared [51]. Another member of lipid microdomains is caveolin-1 which is involved in endocytosis and cell signalling [52]. When caveolin-1 KO mice were infected with *P. aeruginosa*, increased bacterial colonization was observed compared to WT mice with higher mortality rates indicating that caveolin-1 is an important component in fighting infection [52]. Few studies have been conducted to elucidate the role of caveolin-1 in CF. One study has observed that caveolin-1 colocalizes with CFTR and it was found to be increased in CFTR-deficient macrophages inducing an inflammatory phenotype [53]. In sum, the components of microdomains in CF seem to be abnormal, possibly leading to defects in bacteria clearance. 

Ceramides were found to stimulate TLR-4. Following the addition of multiple doses of exogenous C2 and C6 ceramides up to 15 *μ*M, IL-8 was found to be increased in human embryonal kidney cells while cells defective for TLR-4 expression did not display any increase in IL-8 levels. The increases were dose dependent and maximum expression of IL-8 was found following treatment with 1.5 *μ*M C2 and C6 ceramides. Interestingly, IL-8 levels were reduced at higher doses of ceramide treatment. These results indicate that TLR-4 expression is necessary for IL-8 induction by extracellular ceramides. The molecular mechanism regulating the induction of TLR-4 activation by extracellular ceramide was found to differ from that of LPS as TLR-4 activation by ceramide was found to be CD14 independent [54].

A study by Grassme and colleagues showed the importance of ceramide in membrane microdomains and infections with *P. aeruginosa*. The bacteria were shown to interact with sphingolipid membrane platforms following tracheal infection of mice. The disruption of lipid microdomains prevented *P. aeruginosa*-induced apoptosis and induced a 100-fold increase of IL-1*β* gene expression compared to when microdomains remained intact. When aSMase was inhibited, human nasal epithelial cells failed to internalize the bacteria and IL-1*β* release was increased 10-fold compared to when aSMase was active [55]. The addition of C16 ceramide was able to correct the defects seen with aSMase inhibition; internalization, cytokine secretion, and apoptosis returned to normal [55]. This important study demonstrated the role of ceramide in regulating the downstream effects of bacterial infections and gives insight into potential abnormalities in ceramide levels in CF. 

### 3.3. Disregulation of Apoptosis

It is postulated that cells lacking functioning CFTR display defective control of apoptosis [39, 56]. *P. aeruginosa* infection of wild-type epithelial cells was found to induce apoptosis, bacterial internalization, and caspase-3 and -6 activation, while infection of CFTR defective cells did not result in apoptosis [57]. One hypothesis is that CFTR is a transporter of glutathione, an important antioxidant able to protect cells against excessive oxidative bursts. During apoptosis, glutathione is exported out of the cell via CFTR to allow an oxidative stress-induced cell death. In CF, increased levels of intracellular glutathione inhibit the induction of apoptosis. Interestingly, glutathione was found to inhibit nSMase preventing the generation of ceramide following oxidative stress. It has been postulated that low levels of ceramides prevent the completion of apoptosis even when high DNA fragmentation can be observed [58]. 

Ceramides are important messengers in the control of cell death. They activate signalling cascade of proapoptotic molecules such as Akt, Bcl-2, pRB, PKC*α* and in some cell types can activate Raf kinase and JNK [59]. In leukemia cells, increases in TNF*α* can cause DNA fragmentation leading to apoptosis. The addition of C2 ceramide induced DNA fragmentation similar to the one observed in response to TNF*α* increases [60]. Many studies in oncology have involved the modulation of ceramide levels to induce apoptosis [61–63]. Increases in apoptosis of various tumour cells were seen following the treatment of mice with ceramide analogs [64]. Fenretinide treatment was used in cancer studies due to its known induction of apoptosis by increasing intracellular ceramide levels [65] 

It was shown that different species of ceramide were generated following the induction of B-cell receptors leading to apoptosis. C16 ceramide levels were increased within 6 hours after apoptosis induction, while C24 levels were found to peak much later, between 12 to 24 hours after apoptosis induction, when caspases became activated [66]. Following TNF*α*-induction of apoptosis, only one ceramide species, C16, was found to be induced in hepatocytes. Inhibition of ceramide induction by blocking aSMase activity was shown to protect cells from apoptotic cell death. Apoptosis was also reduced in aSMase KO mice [67].

## 4. Ceramide and Disease

The role of ceramide has been investigated in the context of various diseases. In oncology, the role of ceramide as an inducer of apoptosis has been exploited to kill tumour cells in neuroblastoma [68], breast cancer [69], head and neck cancer [70], and colon cancer [71]. Defects in aSMase have been shown to cause Niemann-Pick disease, a progressive neurodegenerative disorder. Knock-out mice for *smpd1*, the gene encoding for aSMase, were found to be resistant to radiation, apoptosis and TNF-*α* due to the reorganization of membrane microdomains following the disruption of ceramide, sphingomyelin, and cholesterol [72]. Interestingly, following infection with *P. aeruginosa*, 90% of knock-out (KO) aSMase mice died while the control mice were able to clear the infection [55]. 

The role of ceramides has also been studied in other neurodegenerative diseases such as amyotrophic lateral sclerosis (ALS) where an accumulation of the sphingolipid was observed in spinal cords of patients and mice. The current hypothesis is that ceramide levels are high due to overactivation of the *de novo* pathway of ceramide synthesis [73]. In Alzheimer's disease, ceramide was linked to the production of amyloid-*β* and to induce plaque formation. Studies have found an upregulation of the enzymes responsible for the synthesis of long chain ceramides such as C18 and C24. Overexpression of aSMase was also observed in certain parts of the brain [74]. IL-1*β* is known to be involved in neurodegeneration at high concentrations in the brain. These high levels were found to be mediated by high levels of ceramide produced by the *de novo* pathway [75]. Increased levels of ceramide in blood plasma were found to be associated with insulin resistance and diabetes. High levels of C18, C20, and C24 : 1 were found in individuals with type 2 diabetes while insulin resistance was associated with low levels of C18, C20, C24, and C24 : 1 [76]. Gaucher's disease is a known monogenic disease of altered lipid metabolism where glycosphingolipids are stored in tissues. Studies have shown that increased macrophage activation and tissue inflammation are involved in the pathology of the disease [77]. Recently a lipidomic study revealed low levels of ceramide in patients with Gaucher's disease [77] indicating that perhaps an impairment in one sphingolipid can alter the metabolism of others.

## 5. Ceramide and Cystic Fibrosis

Contradicting findings have been published about ceramide levels in cystic fibrosis (CF).  Table 1 summarizes the results found about ceramide levels in CF. Our group has observed reduced levels of ceramide in patients with CF. We have analyzed ceramide levels in plasma samples from 10 patients with CF and 10 healthy controls by HPLC/MS/MS. Our results showed that specific species of ceramide were lower in CF patients: C14, C20 : 1, C22, C22 : 1, C24, and also DHC16. When looking at the total levels, CF patients were found to have an overall deficiency in ceramides [8]. We proceeded to analyze total levels of ceramide in TLC purified ceramide fractions using an ELISA based method in plasma of 58 CF patients and 72 controls [4]. Our analysis of ceramide levels in organs affected by CF in our mouse model had also shown defects in CF versus wild-type (WT) mice in plasma, lung, ileum, and pancreas [8, 78]. The pulmonary bacterial load of CF mice is 30-fold higher than WT mice three days post-infection with *P. aeruginosa*. When treated with fenretinide, a semisynthetic retinoid used to increase ceramide in cancer studies [79–82], CF mice were able to clear infection to the same degree as WT mice. Additionally, ceramide levels were increased to the levels observed in WT mice in both plasma and lungs. Mice treated with fenretinide also had a reduction in gene expression of IL-1*β* and S100A8, genes involved in the immune response to bacterial infections. 

Vilela and colleagues demonstrated an increase in IL-8 levels in human tracheal epithelial cells with and without CFTR expression following TNF*α* exposure. The study showed that fenretinide treatment on cells lacking CFTR expression reduced IL-8 levels after TNF*α* stimulation. Using an HPLC method of ceramide detection, they also demonstrated that intracellular levels of ceramide were increased following fenretinide treatment in CFTR-deficient cells [83]. 

Different hypotheses could be made to explain why ceramides are disregulated in CF. Various studies have observed defects in SMases indicating that the recycling pathway in CF is defective. *In vitro* studies by Yu and colleagues have revealed an impairment in the response of aSMase to *P. aeruginosa* in bronchial epithelial cells. Following infection, S9 cells expressing CFTR experienced an increase in intracellular ceramide concentrations whereas IB3-1 CFTR-defective cells did not. Silencing aSMase before infection caused an increase in IL-8 levels in S9 cells. Similarly, when aSMase was added to IB3-1 cells, IL-8 levels were reduced suggesting that aSMase induction and ceramide generation would ameliorate the regulation of cytokine expression in the context of CF. A lower incidence of cellular apoptosis was noticed following infection of CFTR-deficient cells *in vitro* and following *P. aerugonisa* infection of mice which was improved with the addition of aSMase. An increase in pulmonary ceramide levels was observed in normal mice (C57BL/6) mice but not in CF mice (Cftr^tm1unc^-TgN^(FABPCFTR)^) following oropharyngeal instillation of *P. aeruginosa;* however, no differences were seen preinfection [84].

Further *in vitro* studies by Noe and colleagues in endothelial cell models revealed defects in ceramide metabolism. Human microvascular endothelial cells respond to H_2_O_2_ by increasing intracellular ceramide. Pretreating the same cells with an inhibitor of CFTR (CFTR_inh_-172) prevented the increase in ceramide levels. The authors linked the defects in ceramide to the defects observed in apoptosis whereby suggesting that the lack of apoptosis seen in CF may be due to the defect in upregulating ceramide in response to oxidative stress [85]. 

In contrast, studies by the Gulbins group found an accumulation of ceramide in CF. Using first two mouse models of CF (Cftr^tm1unc^-Tg^(FABPCFTR)^ and B6.129P2(CF/3)-Cftr^TgH(neoim)Hgu^), ceramide levels were quantified and by liquid scintillation counting of ceramide spots run on TLC plates compared to C16 ceramide. Additionally, immunochemistry was used on paraffin embedded lungs using the S58-9 antibody. The results showed elevated levels of ceramide in vesicles of epithelial cells from CF mice compared to the appropriate controls. Similar results were obtained using epithelial cells obtained from CF patients by nasal scraping and lung tissue obtained posttransplant. Their study revealed that ceramide accumulated in intracellular lysosomes. The authors attributed this accumulation to the inhibition of aSMase in the lysosomes due to the impairment in acidification of the vesicles. The views on whether pH regulation in vesicles is altered in CF are still conflicting. Certain studies have shown that pH is abnormal in CF vesicles [90] while others claim there are no differences [91, 92]. The authors treated mice with aSMase inhibitors which resulted in the reduction of ceramide levels and improved clearance of bacterial infections [22]. These results agree with the studies by Yu and colleagues mentioned above. When aSMase is not active, ceramide levels do not increase. However, Yu and colleagues observed detrimental effects during bacterial infections when aSMase function is defective while the Gulbins study suggests that inhibition of aSMase enables increased survival of mice infected with *P. aeruginosa*. The same group studied the effects of *P. aeruginosa* infection in macrophages from WT mice and from macrophages obtained from their CF mouse models. Using the DAG kinase assay revealed higher basal levels of ceramide in CF cells compared to WT cells. Using an immunofluorescence-based method, they reported increased ceramide concentrations in WT cells following bacterial infection but not in CF macrophages. In response to the infection, WT cells had increased clustering of ceramide in the plasma membrane but this response was much reduced in CF cells [88].

An immunofluorescence-based method was also used in another study by the same group to evaluate the levels of ceramide in alveoli from explanted lung tissue from CF patients. Concurrent to their previous results obtained with the same methods, they had seen higher staining with anticeramide antibodies in CF tissue compared to donor tissue [87]. No additional methods were used to corroborate their data. Finally, in a fourth study, the group utilized three methods of detection of ceramide: immunofluorescence, DAG kinase assay, and mass spectrometry. The authors looked at three specific species of ceramide: C16, C18, and C20. The levels of each of the three species were not specified in the study but their sum was higher in lungs from their CF mouse models compared to their controls [86]. 

The levels of ceramides in CF were assessed by UK investigators using lung tissues from transplant patients with CF and unused donor lungs. The authors used two methods of ceramide quantification: immunochemistry with the two antibodies (15B4 and S58-9) and HPLC/MS. The immunochemistry results indicated that CF lung tissues had higher median levels of staining compared to all other lung tissue. The authors also found variability in staining between the two antibodies although one was not systematically staining more than the other. Ceramide levels were additionally measured by HPLC/MS although only four species of ceramide were analyzed. It was found that C16, C18, and C20 were elevated in CF lung tissue while no difference was observed for C22 [21]. 

Another study has shown a combination of the two hypothesis finding elevated levels of certain species of ceramide while others being reduced. Using TLC and liquid scintillation counting, ceramide species were analysed in 16HBE14o(-) cells either expressing CFTR or the antisense control gene construct. Four ceramide species were found to be elevated (DHC16, C22, C24, C26) when no CFTR was expressed while 2 species were reduced (C18 and C18 : 1) compared to cells expressing CFTR [89]. 

The question remains why the studies report such different results. The first suggestion is the use of different models either animal or cellular. Different cell types may respond differently to stress, and ceramides may act differently within each cell type. There are many different animal models used in CF research with varying phenotypes. Many attempts to produce an adequate model of CF which would reproduce all phenotypes seen in patients failed due to the lack of lung disease in mice. The first attempts at generating a KO mouse came from the University of North Carolina with the disruption of exon 10 of *Cftr* with neomycin resistance genes [93]. These mice had severe intestinal obstruction but failed to produce any lung disease. A mixed genetic background was at fault and researchers began to backcross the mice to C57BL/6J mice [94]. These mice produce a strong inflammatory pulmonary phenotype without having to induce infections. Reports have described destruction of lung structure [95], thick mucus lining the distal airways [95], infiltration of inflammatory cells around the airways [35], and increases in inflammatory gene expression in the lungs [35]. Additionally, marked pathology in the pancreas was observed with acinar atrophy and increased inflammation [95]. Intestinal phenotypes were also observed with chronic intestinal obstruction. These mice necessitate a liquid diet (Peptamen, Nestle, Brampton, Ontario, Canada) not only to avoid intestinal blockage, a phenotype which can occur in CF patients, but to improve the absorption of nutrients without having to supplement the diet with enzymes. This diet allows CF mice to thrive and to maintain a healthy weight [96]. Other mouse models were generated to express specific *Cftr* mutations such as ΔF508 [97], G551D [98], and G480C [99] but ceramide levels have not been assessed in these models as of yet. 

To escape the need of special diets for CF mice, other models were generated which could express *Cftr* locally in the gut. The Cftr^tm1unc^-Tg^(FABPCFTR)^ express CFTR in the gut under control of a human fatty acid binding protein (FABP) promoter. Although these mice show evidence of impaired alveolar macrophage function without severe intestinal pathology, there is however no description of chronic lung disease in these mice (Jackson Lab site http://www.jax.org/). The B6.129P2(CF/3)-Cftr^TgH(neoim)Hgu^ mouse model has low levels of CFTR expression. These mice can survive on dry pellet food. It is evident that different mouse models present different phenotypes of CF disease (see review [100]). For example, defects in fatty acids such as omega-3 and omega-6 fatty acids have been shown in the B6.Cftr^tm1unc^ KO mouse [2, 4] but not in other models [101]. Some mouse models have failed to demonstrate lung disease and it was speculated that disease modifier genes such as alternate chloride channels could exist in certain mouse backgrounds [94, 102]. It was verified that the B6.Cftr^tm1unc^ KO mouse does not express alternative chloride channels; however, this has not been studied in most mouse models, including the above described Cftr^tm1unc^-Tg^(FABPCFTR)^ [94]. The latter mouse model was generated using a mixed background of 129P3/J, C57BL/6, and FVB/NJ (see http://www.jax.org/).

The issue of diet has been brought up by the Gulbins group as a point contention between *in vivo* ceramide studies [22]. It has been suggested that Peptamen is responsible for the reduced levels of ceramide by elevating cholesterol levels which in turn inhibit aSMase activity [22]. However, the authors overlooked the data from CF patients presented in the same study. The cohort of CF patients assessed in the study was not consuming Peptamen in their diet, were monitored for their cholesterol levels, and none of the patients were placed on a cholesterol reducing diet. The effect of Peptamen on the levels of ceramide was analyzed in our model of CF and WT mice and no influence of Peptamen diet on the level of ceramide was observed [4]. Importantly, these findings were assessed using LC/MS/MS analysis of all detectable species of ceramides which demonstrated that several ceramide species were impaired in CF (both in mice and CF patients) [8]. This deficiency of specific ceramide species was correctable by treatment with fenretinide in mice [8], and we have recently obtained Orphan Drug status approval by the US Food and Drug Administration (FDA) for treatment with fenretinide of CF patients infected with *P. aeruginosa.*

Recently, a genome-wide association study was performed on 4,400 samples collected from five European populations regarding their levels of circulating sphingolipids. The results concluded that there are genetic variations in genes involved in the sphingolipid metabolism indicating that different populations may have varying basal levels of ceramide [103]. This is especially important clinically in CF as genetic variation can affect the severity of the disease, but also in terms of research, differences seen in ceramide levels between studies may be attributed to genetic differences of the populations studied. 

Another point of conflict can arise between the various methods of ceramide analysis. Using antibodies in the detection of ceramides can be misleading as they can bind to a selection of ceramide species and lipids in general. While immunohistochemistry offers an opportunity to visualize staining of tissues, one must be careful to interpret the staining as pure ceramide detection since ceramides are not purified from other lipids which might cross-react with the antibodies. Using the antibodies in ELISAs necessitates an initial separation of lipids by TLC from which the ceramide fraction can be isolated. Without purification, the cross-reactivity of the antibodies prevents a reliable quantification of ceramides. While this assay can reduce costs for the quantification of ceramides for many samples, it cannot provide data on the types of ceramide species nor the concentration of each species present in the sample. DAG kinase assays can be an inexpensive method for ceramide analysis; however, only total levels of ceramide can be assessed and once again, ceramide may not be the only lipid quantified in the end. MS offers unmistakable identification of various species of ceramides; therefore it represents the best solution for accurate quantification. Many are turning to MS as a form of analysis; however, it is questionable why certain studies chose to only look at a small subset of species when it is possible to look at all the known forms of ceramide. The simple explanation would be that the cost per sample using MS is still much greater than the cost for DAG kinase assays and immunodetection. MS requires specialized equipment and knowledge in the technique which may not be available to all laboratories. Regardless, MS is still the most accurate technique currently available for ceramide quantification.

## 6. Conclusion

The area of lipid research is continually gaining momentum. With the improvement of sensitive quantification methods such as MS, it is now possible to accurately measure the levels of ceramides. When testing the levels of ceramides, all detectable species of ceramides (and not only a selection of ceramide species) have to be assessed to fully explore the role of these important mediators in various cellular and molecular mechanisms of gene expression regulation and CF physiology. Ceramides are important in the immune response and it is possible that different species have different roles. The results in the area of CF are contradicting due to the animal models used and methods of ceramide detection. Certain steps can be undertaken to resolve these issues such as the use of internal standards and clear MS protocols should be reported. Most importantly, MS should be made the gold standard for ceramide analysis since the DAG kinase assays and the use of antibodies are known to not be reliable methods. However, despite contracting results, the analysis of ceramide levels remains an important subject to investigate in CF using methods which are likely to generate the most reliable results possible.

## Figures and Tables

**Table 1:** Summary of studies on ceramide in cystic fibrosis.

Author	Model used	Method of analysis	Results
**Studies reporting low levels of ceramide in CF**			
Vilela et al. [83]	Human tracheal epithelial cells	HPLC	Ceramide was found to increase more in cells lacking CFTR than those with functioning CFTR following fenretinide treatment

Guilbault et al. [8]	Plasma from CF patients, and CF mice (B6. Cftr^tm1unc^) Plasma from CF patients and CF mice (B6. Cftr^tm1unc^)	HPLC/MS (13 species of ceramide and 2 dihydroceramide species) TLC followed by ELISA (antibody clone 15B4)	↓ in C14, C20 : 1, C22, C22 : 1, C24, DHC16 in patients ↓in total ceramide levels in patients and mice (plasma and CF related organs). Improvement in bacterial clearance when ceramide is ↑ after fenretinide treatment in CF mice following intratracheal infection with *P. aeruginosa * Correlation between ELISA and MS techniques (*P* = .002, *r* = 0.541, *r* ^2^ = 0.293).

Saeed et al. [78]	CF mice (B6. Cftr^tm1unc^)	TLC followed by ELISA (antibody clone 15B4)	↓in total ceramide levels in mice (plasma and CF related organs).

Guilbault et al. [4]	Plasma from CF patients CF mice (B6. Cftr^tm1unc^)	TLC followed by ELISA (antibody 15B4)	↓ in total ceramide levels in patients and CF mice, correlated with defects in fatty acids

Yu et al. [84]	Bronchial epithelial cells (S9 and IB3-1) Cftr^tm1unc^- Tg^(FABPCFTR)^	HPLC/MS	Ceramide levels are ↓ in cells lacking CFTR compared to control cells during infection.

Noe et al. [85]	Human microvascular endothelial cells	LC/MS (11 species of ceramide and 5 dihydroceramide species)	↑ in ceramide in response to H_2_O_2_ in cells where CFTR is expressed. When CFTR is inhibited, there is no ceramide response.

**Studies reporting high levels of ceramide in CF**			
Teichgräber et al. [22]	CF mice (Cftr^tm1unc^- Tg^(FABPCFTR)^ and B6.129P2(CF/3)- Cftr^TgH(neoim)Hgu^) Human nasal epithelial cells Explanted human lung tissue	Liquid scintillation counting Immunofluorescence (antibody clone S58-9)	↑ in total ceramide levels in CF mice and human cells Blocking aSMase causes a ↓ in ceramide Improvement in bacterial clearance in CF mice when aSMase is inhibited and ceramide levels ↓

Becker et al. [86]	CF mice (Cftr^tm1unc^- Tg^(FABPCFTR)^ and B6.129P2(CF/3)-Cftr^TgH(neoim)Hgu^)	Immunofluorescence (antibody clone S58-9) DAG kinase assay LC/MS (3 species of ceramide)	↑ in total ceramide levels (data represented sum of C16, C18, and C20) Inhibition of aSMase results in ↓ in ceramide and in reduction in susceptibility to intranasal infection with *P. aeruginosa *

Ulrich et al. [87]	Explanted human lung tissue	Immunofluorescence (antibody clone S58-9)	↑ in ceramide in CF lungs

Zhang et al. [88]	Alveolar mouse macrophages	DAG kinase assay Immunofluorescence (antibody clone S58-9)	↑ in total ceramide in CF cells ↑ in ceramide following infection in WT cells, not in CF cells

Brodlie et al. [21]	Explanted human lung tissue	Immunofluorescence (antibody clones S58-9 and 15B4) HPLC/MS (4 species of ceramide)	↑ in total ceramide (observed with staining of antibodies) ↑ in C16, C18, C20. No change in C22.

**Study reporting high and low levels of specific ceramide species in CF**			
Hamai et al. [89]	16HBE14o(-) cells with or lacking CFTR expression	TLC and liquid scintillation counting	↑ in levels of DHC16, C22, C24, C26 ↓ in levels of C18, C18 : 1
